# Targeting Ferroptosis Attenuates Inflammation, Fibrosis, and Mast Cell Activation in Chronic Prostatitis

**DOI:** 10.1155/2022/6833867

**Published:** 2022-06-17

**Authors:** Dongxu Lin, Mengyang Zhang, Changcheng Luo, Pengyu Wei, Kai Cui, Zhong Chen

**Affiliations:** ^1^Department of Urology, Tongji Hospital, Tongji Medical College, Huazhong University of Science and Technology, Wuhan, 430030 Hubei, China; ^2^Institute of Urology, Tongji Hospital, Tongji Medical College, Huazhong University of Science and Technology, Wuhan, 430030 Hubei, China

## Abstract

**Purpose:**

Chronic prostatitis/chronic pelvic pain syndrome (CP/CPPS) is a common urological disorder. Although ferroptosis is closely associated with inflammation, oxidative stress, and neuropathic pain, its role in CP/CPPS has not yet been elucidated. Therefore, we sought to explore the role and mechanism of ferroptosis in the prostatitis development.

**Methods:**

The experimental autoimmune prostatitis (EAP) was established through intradermal immunization of prostate extract. Iron chelator deferoxamine (DFO) and free radical scavenger edaravone (EDA) were applied to evaluate the effects of ferroptosis inhibition on oxidative stress, ferroptosis, inflammation, fibrosis, and mast cell activation in the context of CP/CPPS.

**Results:**

Increased generation of lipid peroxidation products (ROS and MDA) and decreased activities of antioxidant enzymes (SOD and CAT) suggested an aberrant oxidative stress status in EAP model. Elevated iron concentration was observed in the EAP model. Meanwhile, we discovered significant biological performances associated with ferroptosis in CP/CPPS, including the downregulation of the system Xc^−^/GPX4 axis and the upregulation of the ACSL4/LPCAT3 axis. EAP rats performed serious leukocyte infiltration, advanced inflammatory grade, and abnormal expression of inflammatory mediators. Abundant collagen deposition, enhanced RhoA, ROCK1, and *α*-SMA protein levels indicated that EAP rats were prone to suffer from stromal fibrosis compared with control group. An elevated number of degranulated mast cells and corresponding marker TPSB2 represented that mast cell-sensitized pain was amplified in the EAP model. Furthermore, reduction of NRF2/HO-1 indicated a vulnerability of EAP towards ferroptosis response. However, application of DFO and EDA had partially reversed the adverse influences mentioned above.

**Conclusion:**

We first demonstrated that ferroptosis might be a crucial factor of chronic prostatitis progression. Inhibition of ferroptosis using DFO and EDA represented a promising approach for treating prostatitis by ameliorating inflammation, fibrosis, and mast cell activation.

## 1. Introduction

Prostatitis is a common disease in urological clinic, affecting almost 8.2% (2-9%) men and usually recurring with a probability of 20%-50% [1, 2]. It is reported that approximately 35%–50% of men were troubled by chronic prostatitis during the course of their lifetime [1]. Chronic prostatitis/chronic pelvic pain syndrome (CP/CPPS) is the most prevalent phenotypes of prostatitis, manifested by lower urinary tract symptoms, pelvic or genitourinary pain, and possible sexual dysfunction in the absence of urinary infection. Although urinary reflux, oxidative stress, mast cell activation, and psychological factors may partly explain the frequent voiding disorder and pelvic pain [3, 4], the etiology of CP/CPPS still remains controversial.

Ferroptosis, a new nonapoptotic programmed cell death process proposed by Dixon et al. [5], is featured with iron-dependent lipid peroxidation. The inactivation of cysteine/glutamate transporter system Xc^−^ and glutathione peroxidase 4 (GPX4) is responsible for the accumulation of reactive oxygen species (ROS), which was generated by oxidizing polyunsaturated fatty acid (PUFA), and hence triggers the occurrence of ferroptosis. There are growing examples demonstrating that ferroptosis regulates the processes of inflammatory response, oxidative stress, and pathological pain [6, 7], which are the common features of prostatitis patients. Therefore, it is plausible to explore the function of ferroptosis on the progression of CP/CPPS.

Experimental autoimmune prostatitis (EAP) shares similar clinical and pathological characteristics with CP/CPPS and thus is widely accepted as an in vivo model to identify the potential mechanism and treatment of prostatitis. In the current study, iron chelator deferoxamine (DFO) and free radical scavenger edaravone (EDA), two agents that exerted antiferroptosis effects by inhibiting iron overload and lipid peroxidation, respectively, were applied to evaluate the involvement of ferroptosis in the prostatitis.

## 2. Material and Methods

### 2.1. Animals

Adult male Wistar rats (8 weeks old, 200-300 g) were provided by the Laboratory Animal Center of the Tongji Hospital. All rats were housed under same laboratory environment with relative constant temperature (20-26°C), preferable humidity (35%-75%), and regular 12 h light-dark cycle. All procedures involving the animals were approved by the Laboratory Animal Welfare and Ethics Committee of the Tongji Hospital.

### 2.2. Experimental Autoimmune Prostatitis

**Figure 1:**
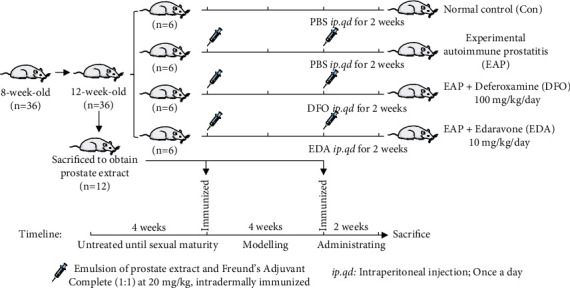
Schematic detailing the process of animal modelling and administrating.

A brief flowchart described the processes of animal modeling and administrating is presented in Figure 1. The induction of EAP model was developed in accordance with Kurita's method [8]. Briefly, adult rats were sacrificed to obtain prostate extracts (PEs). The prostate tissues were homogenized in 0.5% Triton X-100 (Beyotime Biotechnology, Shanghai, China) and centrifuged at 10 000 g for 30 min (4°C). The protein concentrations of supernatants were measured via bicinchoninic acid (BCA) kit (Boster Biological Technology Co. Ltd, Wuhan, China). Then the supernatants were diluted in phosphate buffer solution (PBS) to achieve a final concentration of 40 mg/ml. Subsequently, the tissue homogenates were mixed with complete Freund's adjuvant (CFA; Sigma-Aldrich, MO, USA) in equal volume and intradermal injected into hind footpad and tail base of rats on day 0 and day 28.

We have conducted a preliminary experiment to determine whether PE is sufficient to induce prostatitis. The prostatic specimens of control and EAP groups were applied to observe the histopathological changes using HE staining and to evaluate the inflammatory cell infiltration using immunohistochemistry. The serum levels of proinflammatory cytokines were also detected to assess the systemic cytokine responses between control and EAP groups.

Subsequently, a formal experiment was conducted to explore the role of ferroptosis in chronic prostatitis and the effects of ferroptosis inhibitors on ameliorating inflammation-associated prostatic lesions. 24 rats were randomly categorized into four groups (*n* = 6). The Con group includes rats with sham operation and treated with PBS solution; the EAP group includes rats immunized into the EAP model and treated with PBS solution; the DFO group includes rats immunized into the EAP model and treated with DFO (100 mg/kg/day; MedChemExpress, NJ, USA) solution; the EDA group includes rats immunized into the EAP model and treated with the EDA (10 mg/kg/day; MedChemExpress, NJ, USA) solution. The rat of each group was daily intraperitoneal injection of equal volume solution starting on day 29. We refer to the work of previous experiments to set the dose of DFO and EDA [9, 10]. On day 42, the rats were sacrificed, and the prostate tissues were quickly dissected. A small portion of tissues were fixed with 4% paraformaldehyde for pathological observation, while the remaining ones were frozen at -80°C for further analysis.

### 2.3. Enzyme Linked Immunosorbent Assay (ELISA)

The blood samples were harvested from the carotid artery, and the heparins were added as an anticoagulant. Subsequently, blood samples were centrifuged at 3000 rpm for 15 min (4°C), and the supernatants were collected. The serum concentrations of tumor necrosis factor-*α* (TNF-*α*), interleukin-1*β* (IL-1*β*), and interferon-*γ* (IFN-*γ*) were measured via the rat TNF-*α* ELISA kit (Thermo Fisher Scientific, MA, USA), rat IL-1*β* ELISA kit (Multi Sciences Biotech, Hangzhou, China), and rat IFN-*γ* ELISA kit (Multi Sciences Biotech, Hangzhou, China) according to the manufacturer's protocols.

### 2.4. Measurement of ROS, MDA, SOD, CAT, and Iron Content

Reactive oxygen species (ROS) in the prostate tissue was dyed with 2′,7′-dichlorodihydrofluorescein diacetate (DCFH-DA; MedChemExpress, NJ, USA) probe and visualized under a fluorescence microscope (Olympus, Tokyo, Japan). To detect the oxidative stress degree, the content of lipid peroxidation product malondialdehyde (MDA) and the activities of antioxidant enzymes superoxide dismutase (SOD) and catalase (CAT) were measured with the thiobarbituric acid (TBA) method, xanthine oxidase (XO) method, and ammonium molybdate colorimetry method, respectively (all purchased from Nanjing Jiancheng Bioengineering Institute, Nanjing, China). The tissue iron assay kit (Nanjing Jiancheng Bioengineering Institute, Nanjing, China) was applied to determine the iron content of prostate lysates according to kit instruction. The protein concentration was detected to normalize the data.

### 2.5. Western Blot

The prostate tissues were lysed in the RIPA buffer when protein concentrations were quantified by the BCA kit. Then the equal protein samples (20 *μ*g/lane) were separated by 10% SDS-polyacrylamide gel electrophoresis (SDS-PAGE) and transferred onto polyvinylidene fluoride (PVDF) membranes. After blocking in 3% bovine serum albumin (BSA) solution for 1 h, the PVDF membranes were incubated with antibodies against GPX4 (1 : 1000; ABclonal, Wuhan, China), SLC7A11 (a specific subunit of system Xc^−^) (1 : 1000; ABclonal, Wuhan, China), ACSL4 (1 : 1000; Affinity Biosciences, OH, USA), LPCAT3 (1 : 1000; ABclonal, Wuhan, China), DHODH (1 : 1000; ABclonal, Wuhan, China), COX-2 (1 : 1000; ProteinTech Group, Chicago, USA), IL-2 (1 : 1000; ProteinTech Group, Chicago, USA), IL-6 (1 : 1000; Affinity Biosciences, OH, USA), VCAM-1 (1 : 1000; Abcam, Cambridge, USA), BDNF (1 : 1000; Affinity Biosciences, OH, USA), RhoA (1 : 1000; ProteinTech Group), ROCK1 (1 : 1000; ProteinTech Group), TPSB2 (1 : 1000; ABclonal, Wuhan, China), NRF2 (1 : 500; ABclonal, Wuhan, China), HO-1 (1 : 500; ABclonal, Wuhan, China), and *β*-actin (1 : 1000; ProteinTech Group, Chicago, USA) overnight at 4°C. The membranes were washed thrice in TBST buffer on the following day and incubated with HRP-conjugated goat anti-rabbit or anti-mouse IgG antibody (1 : 5000; Affinity Biosciences, OH, USA) for 1 h, following by another three washing steps. The protein bands were exposed by the enhanced chemiluminescence (ECL) kit (Biosharp, Hefei, China) using the ChemiDoc™ MP Imaging System (Bio-Rad, CA, USA).

### 2.6. Immunohistochemistry

The prostate sections were deparaffinized in xylene and rehydrated in graded alcohols. Next, the tissue slides were immersed in citrate buffer (0.01 mol/L citric acid, pH 6.0) for 20 min and treated with 3% hydrogen peroxide (H_2_O_2_) solution for 10 min. Then the sections were blocked with 10% normal goat serum for 30 min and incubated with antibodies against CD3 (1 : 200; ProteinTech Group, Chicago, USA), CD68 (1 : 200; ProteinTech Group, Chicago, USA), and *α*-SMA (1 : 100; ProteinTech Group, Chicago, USA), respectively, for 1 h. Thereafter, the sections were incubated with biotinylated goat anti-rabbit secondary antibody (1 : 200; ProteinTech Group, Chicago, USA) for 30 min and reacted with 3,3-diaminobenzidine (DAB) for 10 min. Finally, they were counterstained with hematoxylin and observed under a light microscope.

### 2.7. Quantitative Real-Time PCR

TRIzol reagent (Qiagen, CA, USA) was applied to isolate the total RNA of prostate samples following the manufacturer's protocol. The total RNA samples were reverse transcribed into cDNAs. The mRNA expression of genes of interest was determined by quantitative real-time PCR (qPCR) method using the SYBR reagent kit (Takara Bio, Japan) on the QuantStudio™ 6 Flex Real-Time PCR System (Applied Biosystems; Thermo Fisher Scientific, USA). Finally, the relative mRNA expression was calculated using the 2^-*ΔΔ*Ct^ method, and *β*-actin was considered the normalization control.

### 2.8. Histological Examination

The fixed prostate tissues were embedded in paraffin and serially sectioned at a thickness of 5 *μ*m. Then the tissue sections were delivered to hematoxylin-eosin (HE) staining and Masson's trichrome staining for observing morphological changes and collagen deposition, respectively, according to standard procedures.

### 2.9. Inflammatory Grade Score

The inflammation grade was scored according to a four-point grading scale as described in previous studies [11, 12]. In brief, the inflammatory cells were quantified by randomly counting on five spots under high-power fields (×400 magnification). Inflammation classification referred to the following criteria. Grade I is sporadic inflammatory cells in the specific area, and inflammatory cell count is less than 10; Grade II is inflammatory cells aggregation without glandular epithelial tissue destruction or lymphoid nodule/follicle formation, and the inflammatory cell count is 11–20; Grade III is inflammatory cell aggregation with part of glandular epithelial tissue destruction or lymphoid nodule/follicle formation, and the inflammatory cell count is more than 20; Grade IV is inflammatory cell aggregation with mass of glandular epithelial tissue destruction or lymphoid nodule/follicle formation and full spot of inflammatory cells. All the histological evaluations were performed by two independent investigators in a blind manner.

### 2.10. Mast Cell Activation Assessment

The number and activation evidence of mast cells were assessed by toluidine blue staining. That is, the number of mast cells was counted randomly under ×200 magnification. Meantime, the mast cell degranulation was defined as less dense metachromatic granules and/or free granules within the cytoplasm or outside the cell surface, as previously describe [13]. Additionally, tryptase beta II (TPSB2), a marker of mast cell activation, was also detected by the western blot analysis mentioned above.

### 2.11. Statistical Analysis

Statistical analyses were realized via the GraphPad Prism 8 software. The results were expressed as mean ± standard error of mean (SEM), and unpaired Student's *t*-test was applied to compare the difference between groups. *P* < 0.05 was considered statistically significant.

## 3. Results

### 3.1. Immunization of Prostate Extract Induced Inflammatory Responses in the EAP Rat Model

**Figure 2:**
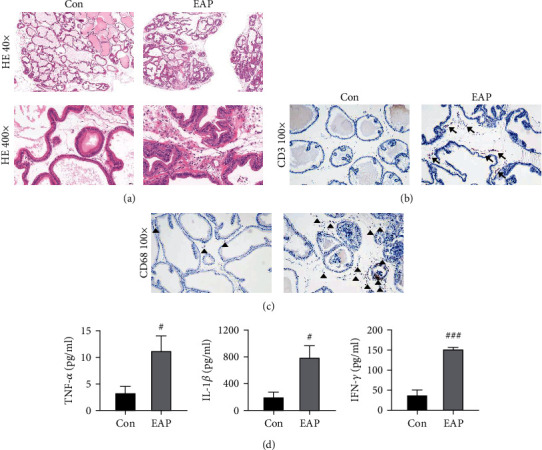
Development of EAP model through immunization of prostate extract. (a) Representative HE-stained histopathological images of the prostate specimens. (b, c) IHC staining for visualizing CD3-positive T cells indicated by arrows and CD68-positive macrophages indicated by triangles in the prostate specimens. (d) The serum contents of proinflammatory cytokines TNF-*α*, IL-1*β*, and IFN-*γ* in the control and EAP groups were determined by ELISA kits. Data was presented as mean ± SEM. ^#^*P* < 0.05 versus the control group; ^###^*P* < 0.001 versus the control group.

To evaluate the feasibility of constructing EAP model by immunization of PEs, we first performed a preliminary experiment to detect the inflammatory responses between control and EAP groups. A significant inflammation-associated prostate lesion was observed in the EAP group, which manifested as diffuse inflammatory cells infiltration, microvascular congestion, and extracellular matrix deposition. The prostate ducts were irregularly shaped, and partial basal membranes were invaded and destroyed by inflammatory cells (Figure 2(a)). The increasing proportions of CD3-positive T cells and CD68-positive macrophages appeared in the prostatic stroma of the EAP model (Figures 2(b) and 2(c)). Meanwhile, serum levels of proinflammatory cytokines TNF-*α*, IL-1*β*, and IFN-*γ* were dramatically increased in the EAP group compared with the control group (Figure 2(d)).

### 3.2. DFO and EDA Ameliorated Oxidative Stress in the EAP Model

**Figure 3:**
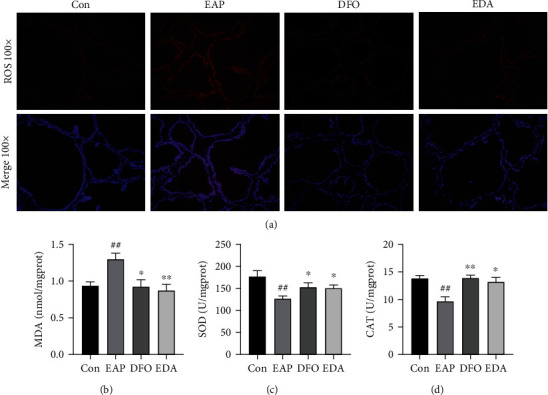
DFO and EDA ameliorated oxidative stress in EAP model. (a) Intercellular ROS generation was evaluated using the fluorescent probe DCFH-DA. (b–d) The MDA level and SOD and CAT activities of prostate lysates were determined using corresponding methods. Results were normalized to protein concentration and presented as mean ± SEM. ^##^*P* < 0.01 versus the control group; ^∗^*P* < 0.05 versus the EAP group; ^∗∗^*P* < 0.01 versus the EAP group.

Generally, the breakdown of redox homeostasis contributes to the accumulation of ROS and hence drives the initiation of oxidative stress. As shown in Figure 3(a), the levels of ROS were significantly attenuated in DFO and EDA groups compared with the EAP group. Besides, the concentration of lipid peroxidation marker MDA and the activities of antioxidant enzymes SOD and CAT were applied to assess the oxidative stress damage in EAP model. The EAP model performed an increased production of MDA and decreased activities of SOD and CAT when compared with the control group. Conversely, DFO and EDA successfully abolished lipid peroxidation and oxidative stress by partially reducing ROS and MDA levels and restoring SOD and CAT activities (Figures 3(b)–3(d)).

### 3.3. DFO and EDA Attenuated Ferroptosis in EAP Model

**Figure 4:**
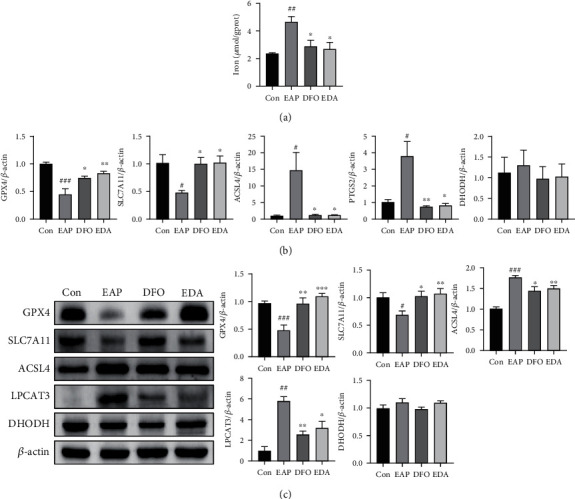
DFO and EDA attenuated ferroptosis in EAP model. (a) The iron concentration of prostate lysates was determined by the commercial kit. Results were normalized to protein concentration. (b) The mRNA levels of ferroptosis biomarkers GPX4, SLC7A11, ACSL4, PTGS2, and DHODH relative to internal control were determined by the RT-PCR method. (c) The protein levels of ferroptosis biomarkers GPX4, SLC7A11, ACSL4, LPCAT3, and DHODH were determined by the western blot method. The relative quantification result of each band was performed relative to *β*-actin. Data was presented as mean ± SEM. ^#^*P* < 0.05 versus the control group; ^##^*P* < 0.01 versus the control group; ^###^*P* < 0.001 versus the control group; ^∗^*P* < 0.05 versus the EAP group; ^∗∗^*P* < 0.01 versus the EAP group; ^∗∗∗^*P* < 0.001 versus the EAP group.

Considering the robust evidences implying that inflammation and oxidative stress are positively associated with ferroptosis, we aimed to delineate the mechanisms underlying ferroptosis in prostatitis progression. Iron, an essential driver of ferroptosis, was elevated markedly in the EAP group compared with the control group. The medication of iron chelator DFO and free radical scavenger EDA, two agents with ferroptosis inhibitory capacity, has obviously reduced the iron content (Figure 4(a)). The mRNA expression of GPX4 and SLC7A11/Xc^−^ was downregulated, and the mRNA expression of ACSL4 and PTGS2 was upregulated in the EAP group when compared with those in the control group (Figure 4(b)). In line with the transcription level, the protein levels of GPX4 and SLC7A11/Xc^−^ were also inhibited in EAP model, while those of ACSL4 and LPCAT3 were performed in an opposite manner (Figure 4(c)). However, the expression of ferroptosis-associated indicators mentioned above was significantly reversed in the presence of DFO and EDA treatment. No changes were found in DHODH mRNA and protein levels among four groups.

### 3.4. DFO and EDA Alleviated the Inflammatory Responses in the EAP Model

**Figure 5:**
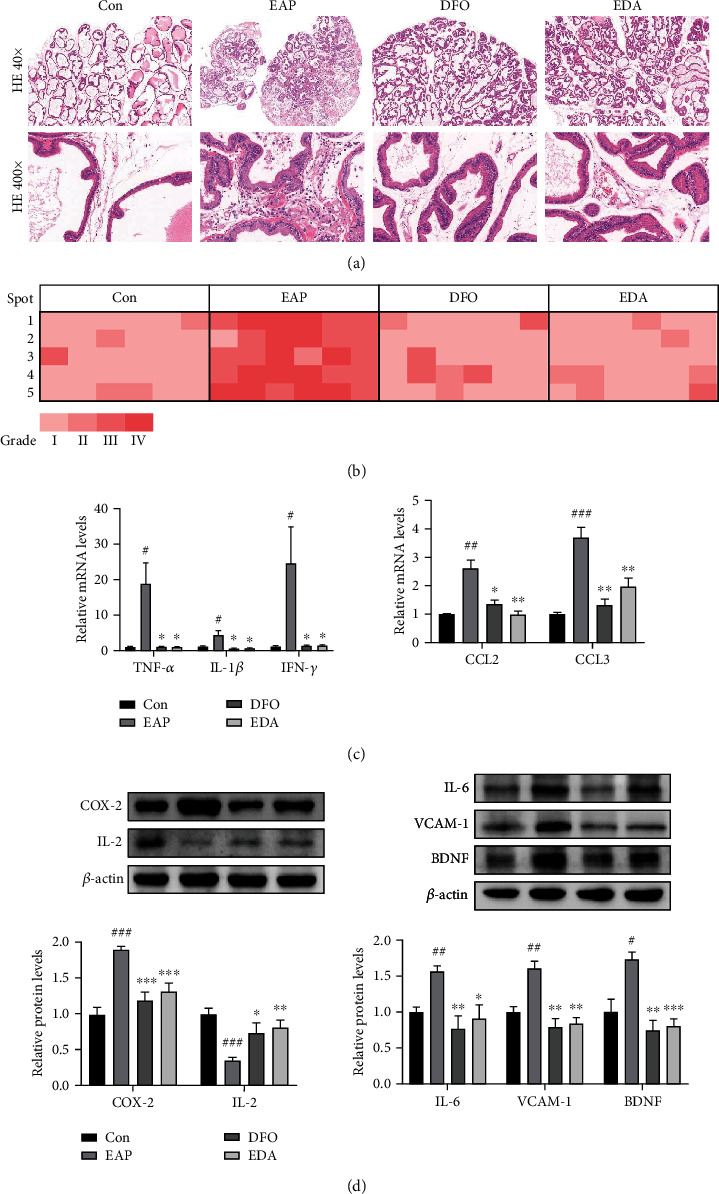
DFO and EDA alleviated the inflammatory responses in EAP model. (a) Representative HE-stained images of prostate tissues. (b) Inflammation grades for prostate samples were scored on the basis of HE sections (×400 magnification). The columns represent five random spots of one rat, while the rows denote six animals involved in each group. (c) The mRNA expression of proinflammatory cytokines (TNF-*α*, IL-1*β*, and IFN-*γ*) and chemokines (CCL2, CCL3) relative to *β*-actin was performed by the RT-PCR method. (d) The protein levels of immunological mediators (COX-2 and IL-2) and cytokines (IL-6, VCAM-1, and BDNF) were performed using the western blot method, and the relative quantitative results are shown. Data was presented as mean ± SEM. ^#^*P* < 0.05 versus the control group; ^##^*P* < 0.01 versus the control group; ^###^*P* < 0.001 versus the control group; ^∗^*P* < 0.05 versus the EAP group; ^∗∗^*P* < 0.01 versus the EAP group; ^∗∗∗^*P* < 0.001 versus the EAP group.

Compared with control group, the prostate tissues of EAP model showed intensive immunological responses, characterized by diffusely leukocyte infiltration, partially basilar membrane interruption, small vascular proliferation and interstitial fibrogenesis, which were remarkably alleviated after DFO and EDA treatment (Figure 5(a)). The inflammatory grade score was applied to assess the histopathological alteration and inflammation severity, which showed that DFO and EDA could effectively decrease inflammatory indexes (Figure 5(b)). In addition, the elevated mRNA expression of proinflammatory cytokines (TNF-*α*, IL-1*β*, and IFN-*γ*) and chemokines (CCL2 and CCL3) were rescued significantly when treated with DFO and EDA (Figure 5(c)). Meanwhile, the upregulation of inflammatory mediator COX-2 and downregulation of immunomodulatory factor IL-2 were reversed obviously in the presence of DFO and EDA, while the increased production of cytokines in the EAP group, including IL-6, VCAM-1, and BDNF, was significantly reversed in the DFO and EDA groups (Figure 5(d)).

### 3.5. DFO and EDA Prevented Stromal Fibrosis in the EAP Model

**Figure 6:**
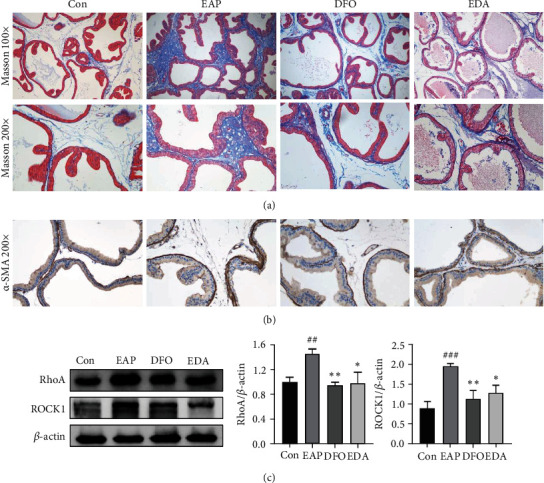
DFO and EDA prevented stromal fibrosis in the EAP model. (a) Representative Masson's trichrome-stained images of prostate tissue. (b) Immunohistochemical staining of *α*-SMA. (c) Western blot analysis of RhoA/ROCK1 signaling, and the relative quantitative results are shown. Data was presented as mean ± SEM. ^##^*P* < 0.01 versus the control group; ^###^*P* < 0.001 versus the control group; ^∗^*P* < 0.05 versus the EAP group; ^∗∗^*P* < 0.01 versus the EAP group.

Masson's trichrome staining uncovered that DFO and EDA could prevent the abundant collagen deposition of PE-induced prostatitis (Figure 6(a)). To further validate the curative effects of DFO and EDA, the expression of RhoA/ROCK1 signaling and myofibroblast marker *α*-SMA were determined by the western blot and immunohistochemistry method. It was revealed that these agents had greatly avoided the activation of the RhoA/ROCK1 pathway and *α*-SMA expression in the EAP model (Figures 6(b) and 6(c)).

### 3.6. DFO and EDA Suppressed Mast Cell Activation in the EAP Model

**Figure 7:**
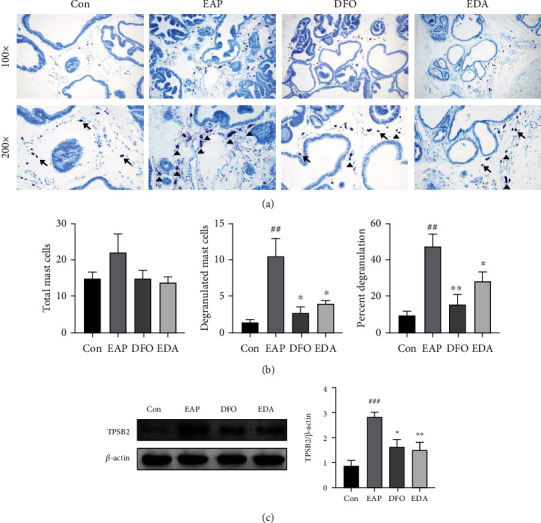
DFO and EDA suppressed mast cell activation in EAP model. (a) Toluidine blue staining to visualize mast cells in the prostate tissues. (b) The number of total, activated mast cells, and the percentages of activated ones were counted on the basis of toluidine blue staining under ×200 magnification. The arrows indicate the silent (nongranulated) mast cells with intact cytomembrane, while the triangles indicate the activated (granulated) mast cells with diffuse metachromatic granules. (c) Western blot analysis of TPSB2 to reflect mast cell degranulation level in the prostate tissue. Relative quantification of TPSB2 was visualized. Data was presented as mean ± SEM. ^##^*P* < 0.01 versus the control group; ^###^*P* < 0.001 versus the control group; ^∗^*P* < 0.05 versus the EAP group; ^∗∗^*P* < 0.01 versus the EAP group.

Given several evidences suggesting a pivotal role of mast cell in driving pain of prostatitis, we next sought to elucidate whether DFO and EDA contribute to suppression of mast cell activation. Mast cell was identified by toluidine blue staining, and the number of total and degranulated cells was recorded. Our results witnessed an insignificant slight increase of mast cell in the EAP group. However, a significantly increasing number and percentage of activated/degranulated mast cells were observed in EAP group, which were ameliorated by DFO and EDA (Figures 7(a) and 7(b)). The suppression of mast cell tryptase (TPSB2), an indicator of mast cell degranulation, also confirmed the therapeutic potential of DFO and EDA in inhibiting mast cell activation of prostatitis (Figure 7(c)).

### 3.7. DFO and EDA Inhibited EAP-Associated Ferroptosis through Restoring the NRF2/HO-1 Pathway

**Figure 8:**
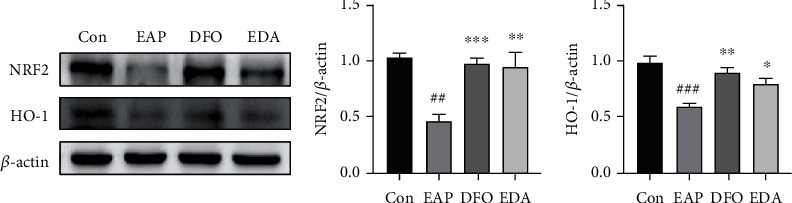
DFO and EDA inhibited EAP-associated ferroptosis through restoring the NRF2/HO-1 pathway. Western blot analysis was applied to assess the protein expression of the NRF2/HO-1 axis. ^##^*P* < 0.01 versus control group; ^###^*P* < 0.001 versus the control group; ^∗^*P* < 0.05 versus the EAP group; ^∗∗^*P* < 0.01 versus the EAP group; ^∗∗∗^*P* < 0.001 versus the EAP group.

To further explore the mechanism of ferroptosis on PE-induced prostatitis, we investigated the alteration of NRF2-antioxidant response element (ARE) signaling. We found that NRF2 and HO-1 protein levels were dramatically suppressed in the EAP group and significantly elevated in the DFO or EDA group, indicating the curative effects of DFO and EDA on prostatitis were likely to mediated by activating NRF2/HO-1 signaling (Figure 8).

## 4. Discussion

Since autoimmunity is the potential etiology of CP/CPPS, the EAP model is considered a preferable animal model to explore the pathological mechanisms and molecular biological characteristics of CP/CPPS and is widely applied to discover novel therapeutical strategies [8, 14]. The EAP model was generally established by immunization of various autoantigens, including PEs, male accessory gland (MAG) extracts, prostatic steroid-binding protein (PSBP), and T2 peptide [15, 16]. Our previous study had established the EAP model through intradermal immunization of PEs [17]. In the current study, the EAP model was developed in the similar method. It was shown that the prostate specimen of the EAP group performed intensive inflammatory responses, including extensive inflammation lesions, increased number of inflammatory cells such as CD3-positive T cells and CD68-positive macrophages, and elevated serum concentrations of proinflammatory cytokines such as TNF-*α*, IL-1*β*, IFN-*γ*. These findings support the notion that EAP animal could simulate pathological features of CP/CPPS patients.

Ferroptosis has been demonstrated to implicate in a lot of nonbacterial inflammatory disorders, such as experimental autoimmune encephalomyelitis, nonalcoholic steatohepatitis, and osteoarthritis [18–20]. In the current study, we found that the EAP group was susceptible to ferroptosis due to the condition of iron overload and lipid peroxidation. Inhibition of ferroptosis was sufficient to mitigate EAP-associated inflammatory reaction, stromal fibrosis, and mast cell activation. Specifically, ROS accumulation, MDA rising, and SOD and CAT depletion inferred an aberrant oxidative stress condition in experimental rats. Moreover, elevated iron concentration and differential expression of ferroptosis indicators, including GPX4, SLC7A11/Xc^−^, ACSL4, LPCAT3, and PTGS2, implied that ferroptosis engaged in the development of prostatitis in the context of iron overload and oxidative damage. Moreover, exacerbated inflammatory infiltration, enhanced expression of proinflammatory cytokines (TNF-*α*, IL-1*β*, and IFN-*γ*) and chemokines (CCL2, CCL3), abnormal production of immunological mediators (COX-2 and IL-2), and elevated generation of cytokines (IL-6, VCAM-1, and BDNF) were observed in prostatitis models. Increased deposition of collagen fiber, in coordination with activation of RhoA/ROCK1 signaling and *α*-SMA expression confirmed the existence of stromal fibrosis. The activation of mast cell was also demonstrated by morphological staining and marker immunoblotting. What is more, the impairment of NRF2/HO-1 signaling might attribute to the vulnerability towards ferroptosis. Excitingly, parameters mentioned above were reversed by iron chelator DFO and free radical scavenger EDA, suggesting a feasibility of ferroptosis-targeted therapy on prostatitis.

**Figure 9:**
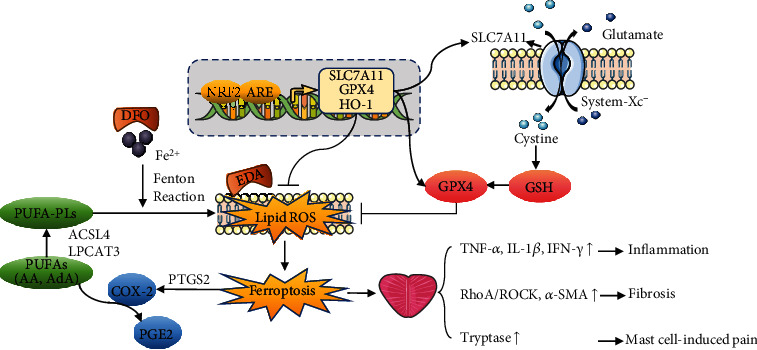
Summary of the mechanism of ferroptosis in chronic prostatitis. Cystine is intracellular transported through system Xc^−^ and utilized for GSH synthesis, which is an essential cofactor of GPX4 for removing phospholipid hydroperoxides (PLOOHs). ACSL4 and LPCAT3 are involved in the esterification and incorporation process of PUFA into membrane lipid and thus served as the substrates of lipid peroxidation. Iron overload initiated the Fenton reaction to generate excessive lipid ROS, including PLOOH, through oxidizing PUFAs or PUFA-containing lipids. The excessive production of ROS and suppression of GPX4 reduced host defense against oxidative stress and thus mediated the onset of ferroptosis in prostatitis. Ferroptosis-associated cell breakdown brought the recruitment of macrophage cell, T cell, and mast cell, resulting in intensive inflammation and pain sensitization. The uncontrolled inflammatory response promotes the occurrence of oxidative damage and ferroptosis in a feedback manner and therefore causes persistent inflammation, fibrosis, and pain in patients with chronic prostatitis. NRF2 positively regulated antioxidant molecules (SLC7A11, GPX4, HO-1, etc.) to inhibit ferroptosis. Iron chelator DFO and free radical scavenger EDA prevent ferroptosis by reducing iron availability and lipid peroxidation.

Ferroptosis was considered to possess three essential hallmarks characterized by the deficiency of lipid peroxide repair enzyme GPX4, the easy availability of redox-active iron, and the oxidation of PUFA. In this study, the inactivation of the system Xc^−^/GPX4 axis, accumulation of iron, and activation of ACSL4/LPCAT3 axis strongly highlighted the important role of ferroptosis on prostatitis development. The occurrence of ischemia-reperfusion injury and oxidative stress in prostatitis patients might be the initiating factors of ferroptosis [21, 22]. That is, the destruction of vessel promoted the accumulation of iron, while the disruption of redox biology contributed to oxidative damage. In the Fenton reaction, iron induced phospholipid peroxidation through reacting with H_2_O_2_ to generate hydroxyl radical. The excessive production of lipid ROS and suppression of GPX4 reduced host defenses against oxidative stress and thus mediated the onset of ferroptosis in prostatitis. After execution of ferroptosis, the cell fragments caused the recruitment of inflammatory cells, including macrophage and Th1 cell, to exacerbate inflammatory cascades via producing proinflammatory cytokines TNF-*α*, IL-1*β*, and IFN-*γ*. These cytokines then promoted the secretion of tryptase, histamine, and prostaglandin E2 (PGE2) from activated mast cell and therefore caused the prolonged pain in chronic prostatitis patients. Meanwhile, the persistent inflammatory reaction was sentenced to the progression of fibrogenesis with the activation of RhoA/ROCK1 signaling, which contributed to the fibroblast-to-myofibroblast transdifferentiation. Interestingly, DHODH, a newly identified ferroptosis defense molecular [23], performed no change in EAP model, which may attribute to its predominant function in cell with low GPX4 expression. By the way, PTGS2 is considered a specific biomarker of ferroptosis, and its encoded protein COX-2 can catalyze the metabolism of PUFAs, especially arachidonic acid (AA), into prostaglandin isoforms PGE2, thereby bridging the cross talk between pain and ferroptosis in prostatitis [24, 25]. The aggravation of iron-dependent Fenton reaction and deficiency of antioxidant capacity indicate possible curative effects of iron chelator DFO and free radical scavenger EDA on ferroptosis-associated diseases. Encouragingly, with the assistance of DFO and EDA, the restoration of the system Xc^−^/GPX4 pathway, suppression of the ACSL4/LPCAT3 pathway, and reduction of ROS accumulation conferred the resistance to ferroptosis in prostate tissue (Figure 9). Further studies are required to identify whether administration of ferroptosis activator could induce the occurrence of prostatitis phenotype.

Nuclear factor erythroid 2-related factor 2 (NRF2) is a vital transcription factor required for the maintenance of redox homeostasis [26]. In response to specific stimuli, NRF2 will translocate into nucleus to target the transcription of ARE-regulated genes which are engaged in iron homeostasis (HO-1, FTH1), GSH synthesis (SLC7A11, GCLC/GLCM), and redox regulation (GPX4, NQO1) [27–29]. Previous studies found that NRF2 was essential for the activation of HO-1, GST, and NQO1, which contributed to the suppression of inflammatory response and oxidative stress with the reduction of cytokines (TNF-*α*, IL-1*β*) and lipid peroxidation (ROS and MDA), and improvement of antioxidant activity (SOD, CAT) in prostatitis [30–32]. Our current study further confirmed that DFO and EDA exerted antiferroptosis effects on prostatitis model through regulating the NRF2/HO-1 pathway, providing a novel insight into prostatitis progression and treatment. However, blockage or depletion of NRF2 protein is also required to validate the detailed mechanism of NRF2 signaling in prostatitis progression in the further work.

Several antioxidants and free radical traps have performed therapeutical potential towards prostatitis by preventing lipid peroxidation and oxidative damage to correct the host homeostasis, such as N-acetylcysteine and *α*-tocopherol [30, 33]. Selenium, an essential component of GPX4 that confer resistance to ferroptosis, has been clinically applied in treating chronic prostatitis through inhibiting oxidative stress and inflammatory response [34]. Currently, DFO and EDA were effective in treating prostatitis with the capacities of iron chelating and free radical neutralizing. Consistent with our results, acting as a ferroptosis inhibitor, DFO performed potent antiferroptosis capacity mainly through depleting iron, suppressing ROS-mediated oxidative damage, and restoring GPX4-governed lipid peroxidation repair system [9, 35]. Furthermore, DFO had also exerted favorable inhibitory activities against inflammation, fibrosis, oxidative stress, and neuropathic pain [36–38]. In regards to EDA, it was responsible for the suppression of inflammation and oxidative damage statuses by inhibiting MDA production, enhancing SOD activity, and decreasing the productions of TNF-*α* and IL-1*β* in cerebral focal ischemia and the cadmium-exposed toxicity model [39, 40]. The combination of EDA and NO appeared to prevent ROS-induced mast cell degranulation [41]. Taking together, DFO and EDA performed preferable efficacy in CP/CPPS treatment via inhibiting ferroptosis, which is responsible for the alleviation of inflammation, fibrosis, and mast cell activation.

## 5. Conclusion

Overall, our results demonstrated that DFO and EDA brought great benefits to prostatitis treatment via inhibiting ferroptosis, accompanied by the reduction of inflammation, fibrosis, and mast cell activation, which was partly attributed to the reactivation of the NRF2/HO-1 signaling. Therefore, we proposed the assumption that inhibition of ferroptosis through administrating DFO and EDA represented an attractive approach for treating prostatitis.

## Data Availability

The data used to support the findings of this study are included within the article. If any other data are needed, please contact the corresponding author.
